# Supplementary material for: Composition, Color Stability and Antioxidant Properties of Betalain-Based Extracts from Bracts of Bougainvillea
Source: Molecules. 2022 Aug 11;27(16):5120. doi: 10.3390/molecules27165120 (PMC9412980; doi:10.3390/molecules27165120)
Supplement: Supplementary file 1 [file molecules-27-05120-s001.zip › molecules-1833320-supplementary.pdf]

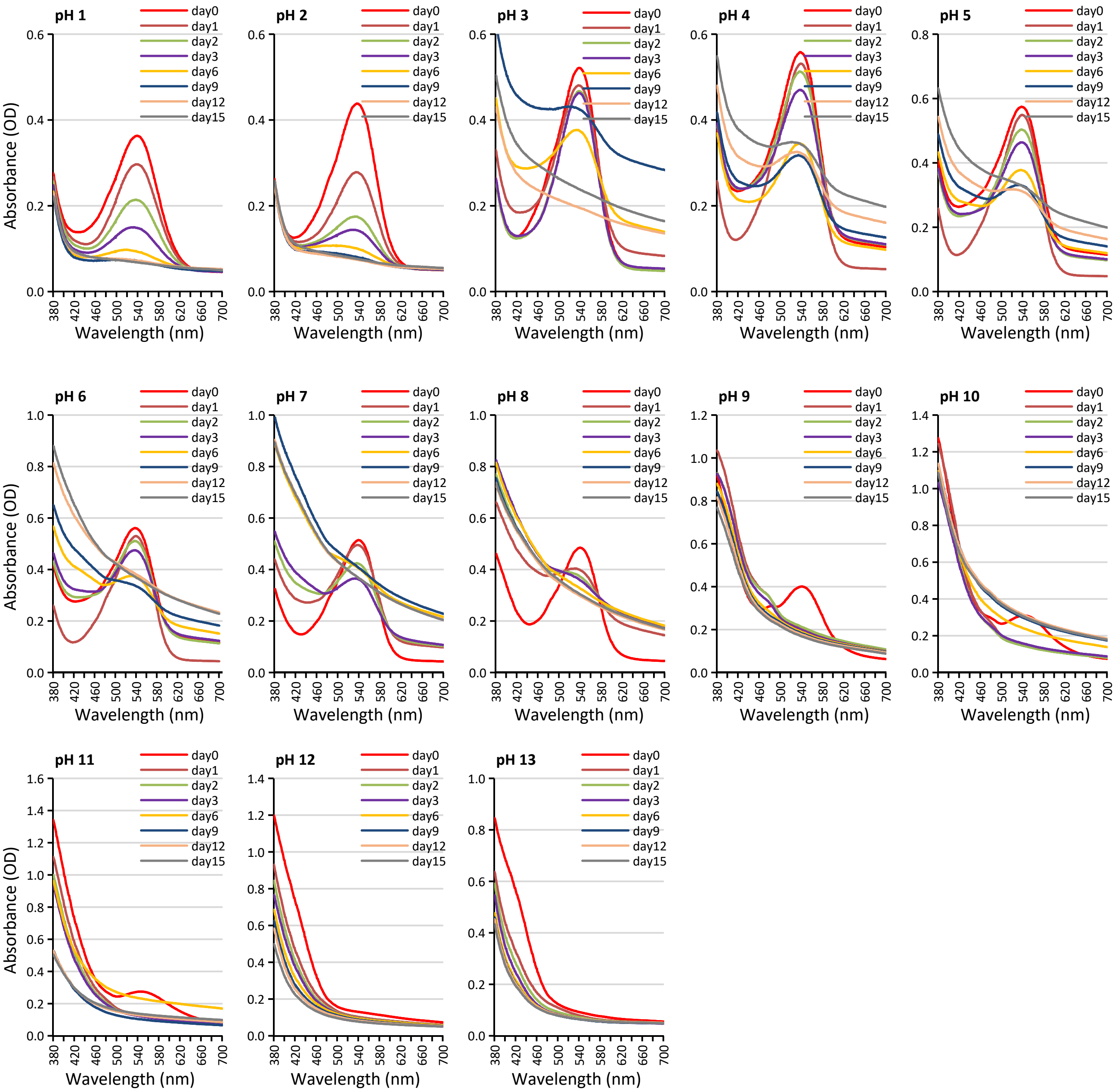

Figure S1. Spectra for betalain degradation in "3 Mature Purple" at different pHs.

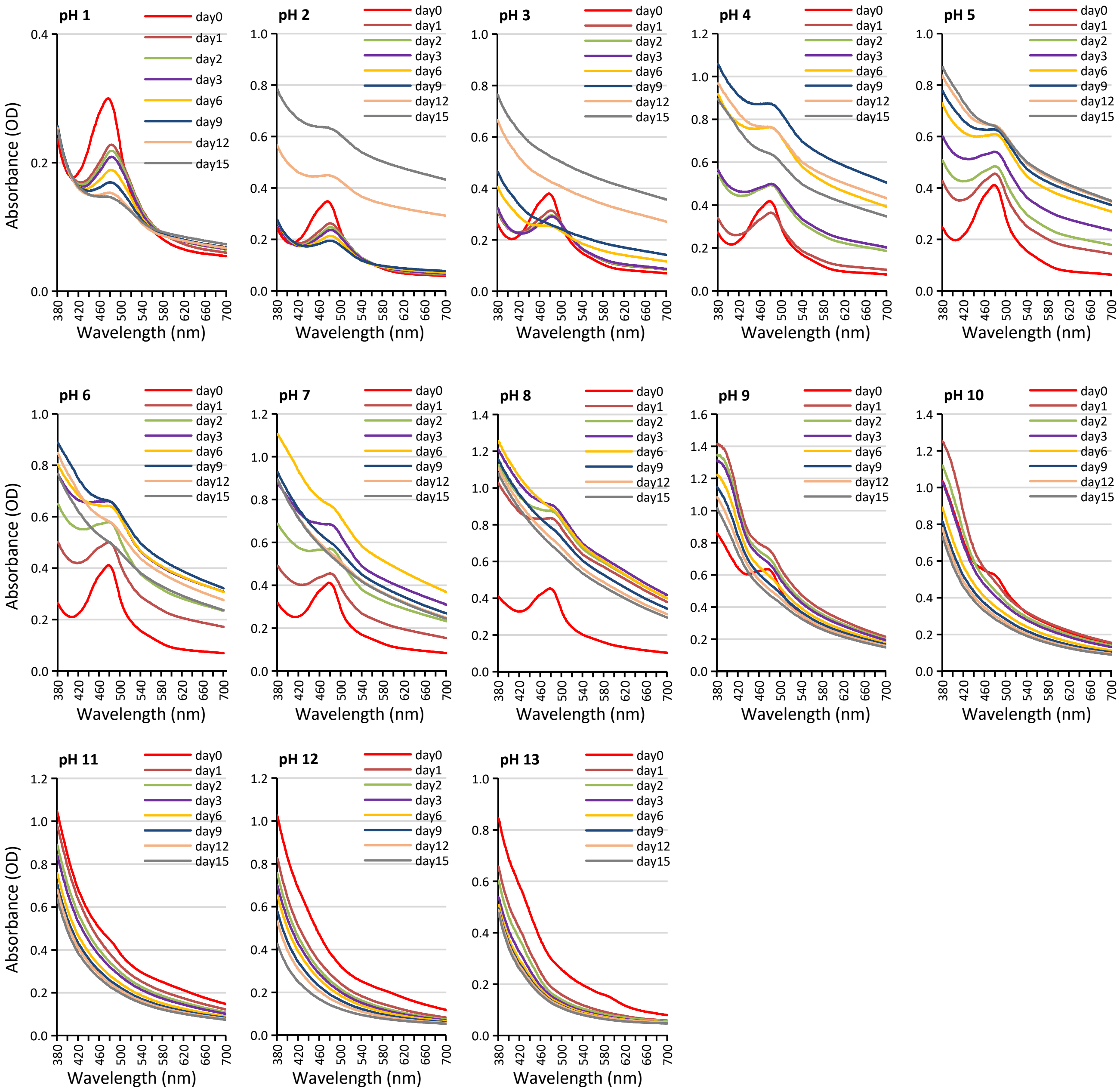

Figure S2. Spectra for betalain degradation in “7 Orange” at different pHs.

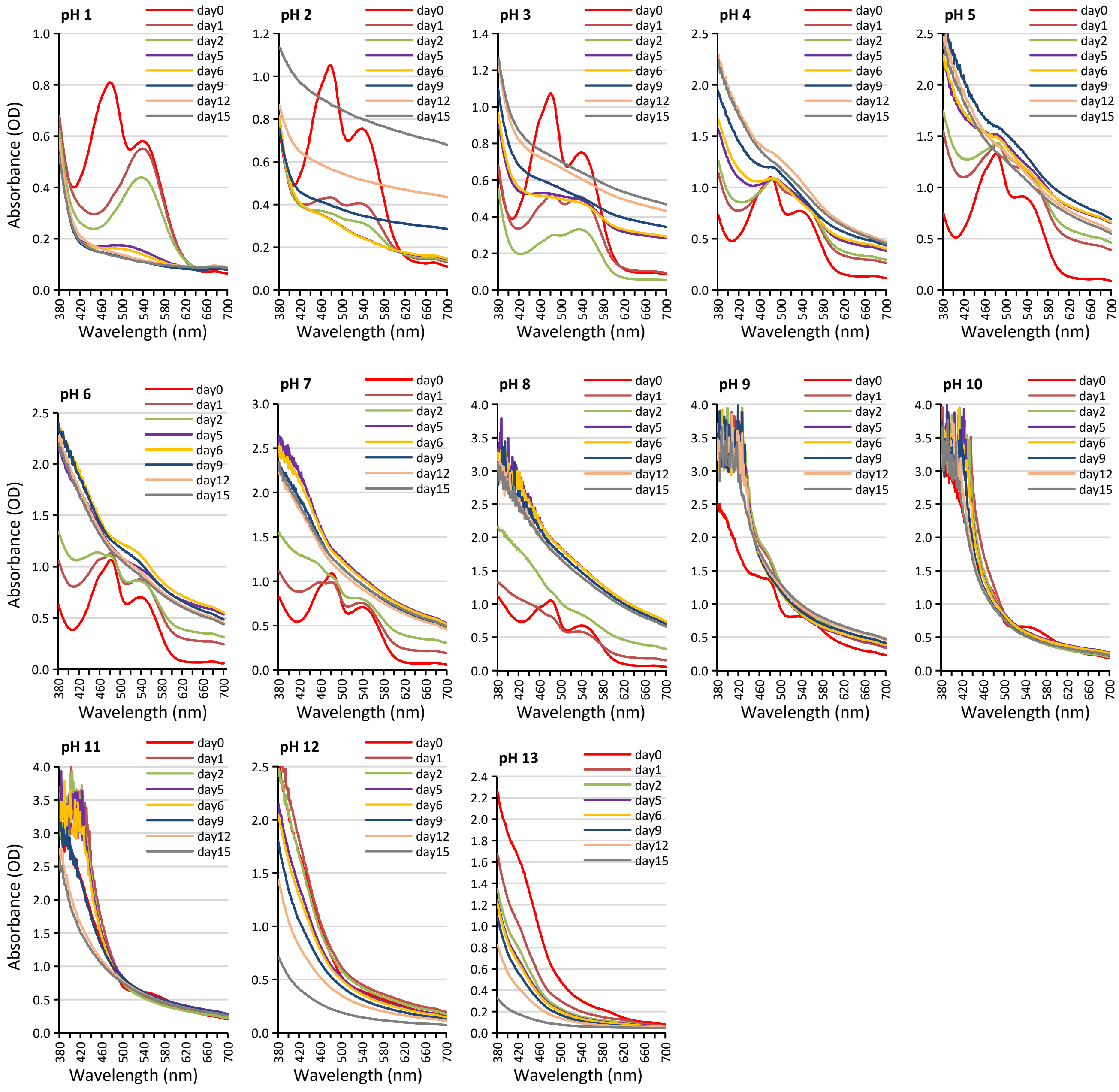

Figure S3. Spectra for betalain degradation in "4 Young red" at different pHs.
